# The Effects of Antigen-Specific IgG1 Antibody for the Pulmonary-Hypertension-Phenotype and B Cells for Inflammation in Mice Exposed to Antigen and Fine Particles from Air Pollution

**DOI:** 10.1371/journal.pone.0129910

**Published:** 2015-06-16

**Authors:** Sung-Hyun Park, Wen-Chi Chen, Nedim Durmus, Bertram Bleck, Joan Reibman, Gabriela Riemekasten, Gabriele Grunig

**Affiliations:** 1 Department of Environmental Medicine, New York University Langone Medical Center, Tuxedo, New York, United States of America; 2 Department of Medicine, Division of Pulmonary Medicine, New York University Langone Medical Center, New York, New York, United States of America; 3 Clinic for Rheumatology, University of Lübeck, Lübeck, Germany; University Hospital Freiburg, GERMANY

## Abstract

Air pollution is known to exacerbate chronic inflammatory conditions of the lungs including pulmonary hypertension, cardiovascular diseases and autoimmune diseases. Directly pathogenic antibodies bind pro-inflammatory cell receptors and cause or exacerbate inflammation. In contrast, anti-inflammatory antibody isotypes (e.g. mouse immunoglobulin G1, IgG1) bind inhibitory cell receptors and can inhibit inflammation. Our previous studies showed that co-exposure to antigen and urban ambient particulate matter (PM_2.5_) induced severe pulmonary arterial thickening and increased right ventricular systolic pressures in mice via T-cell produced cytokines, Interleukin (IL)-13 and IL-17A. The aim of the current study was to understand how B cell and antibody responses integrate into this T cell cytokine network for the pulmonary hypertension phenotype. Special focus was on antigen-specific IgG1 that is the predominant antibody in the experimental response to antigen and urban ambient PM_2.5_. Wild type and B cell-deficient mice were primed with antigen and then challenged with antigen and urban particulate matter and injected with antibodies as appropriate. Our data surprisingly showed that B cells were necessary for the development of increased right ventricular pressures and molecular changes in the right heart in response to sensitization and intranasal challenge with antigen and PM_2.5_. Further, our studies showed that both, the increase in right ventricular systolic pressure and right ventricular molecular changes were restored by reconstituting the B cell KO mice with antigen specific IgG1. In addition, our studies identified a critical, non-redundant role of B cells for the IL-17A-directed inflammation in response to exposure with antigen and PM_2.5_, which was not corrected with antigen-specific IgG1. In contrast, IL-13-directed inflammatory markers, as well as severe pulmonary arterial remodeling induced by challenge with antigen and PM_2.5_ were similar in B cell-deficient and wild type mice. Our studies have identified B cells and antigen specific IgG1 as potential therapeutic targets for pulmonary hypertension associated with immune dysfunction and environmental exposures.

## Introduction

Pulmonary hypertension significantly decreases quality of life and shortens life expectancy [1–3]. In pulmonary hypertension, the increases in the pulmonary pressure are associated with the remodeling of the pulmonary arteries [1] and structural and metabolic changes in the right ventricle of the heart [4].

Environmental exposures can precipitate pulmonary hypertension [5, 6]. Silicosis (coal miner and stone worker disease) was a cause of pulmonary hypertension in the US and Western Europe in the early 20th century [7], with the first described cases in 1846 [8]. Pulmonary hypertension induced by exposure to silica is still a major problem particularly in Asia and South America [9]. Cigarette smoke exposure is thought to be the most important trigger of pulmonary hypertension in chronic obstructive pulmonary disease [10]. Morphologic changes in the right heart (greater right ventricular mass and end-diastolic volume) are associated with the intensity of traffic related air pollution (as measured by outdoor nitric oxide concentration) [11]. In addition, environmental exposures to silica or organic chemicals can exacerbate autoimmune diseases, including systemic sclerosis [12], and environmental exposures can cause autoimmune alterations of the immune system [13]. Autoimmune disorders such as systemic sclerosis and systemic lupus erythematosus [14], in turn, are significant risk factors for the development of pulmonary hypertension.

Our group has recently shown that exposure of immunized mice with a weak antigen that induces T helper (Th)2 responses results in severe thickening of approximately a quarter of the pulmonary arteries [15]. We then increased the intensity of airway exposure by co-administering antigen and particulate matter 2.5 (PM_2.5_ collected from urban air). In that case, the percentage of severely thickened arteries in the lungs and the right ventricular systolic pressure were significantly increased [5]. Our studies further focused on the signature cytokines of Th2 and Th17 responses, Interleukin (IL)-13 and IL-17A respectively. The data showed that IL-13 and IL-17A were together necessary for the increase in right systolic ventricular pressure induced by co-exposure to antigen and PM_2.5_ [16]. In addition, our data identified cellular and molecular response arms that were controlled by either IL-13 or IL-17A in the lungs of animals exposed to an antigen and PM_2.5_ [16].

Increased autoantibody levels are commonly detected in pulmonary hypertension associated with autoimmune diseases [17–19]. In an animal model of toxicosis induced by the plant pyrrolizidine alkaloid monocrotaline, an increased titer of autoantibodies to pulmonary vascular cells was seen following the development of pulmonary hypertension [20]. In this study, repeated injections of control wild type animals with auto-antibody containing plasma or enriched immunoglobulins was sufficient to produce the vascular remodeling and an increase in the right ventricular systolic pressure [20]. B cells that have escaped the tolerance-selection process or that have been inappropriately activated produce the pathogenic auto-antibodies [13]. The auto-antigen specificity of the B cell response and the pathogenic autoantibodies has been subject of much research [21–31]. However, the role of the isotype of the pathogenic antibodies has received less attention. Antibody isotypes are important because they determine complement activation, Fc receptor binding and subsequent cellular and inflammatory responses [32]. For example, complement-activating antibodies (IgM or mouse IgG2a/c) when complexed with antigen induce type III hypersensitivity with neutrophilic infiltration and vasculitis [33] that can result in vascular remodeling. The mouse IgG2a/c antibodies also bind activating Fc receptors, further exacerbating the inflammation by activating neutrophils, macrophages and dendritic cells [34, 35]. In contrast, antibodies that do not activate complement, do not bind activating Fc receptors, and instead bind inhibitory Fc receptors (e.g. mouse IgG1) can be inhibitors of inflammation [32, 36–39].

Currently, much interest is directed at understanding the immune mediators (cytokines, T cells, B cells, antibodies) which can cause or exacerbate pulmonary hypertension diseases [17–19]. However, little is known how these mediators are linked and which independent roles they may have. Previously, we have shown that the increase in right ventricular systolic pressure (RVSP) induced by exposure to antigen and PM_2.5_ is significantly ameliorated by simultaneously blocking the T helper (Th) 2 and Th17 mediators, Interleukin (IL)-13 and IL-17A [16]. The current studies were aimed to understand how B cell and antibody responses integrate into the cytokine network for the pulmonary hypertension phenotype. Special focus was on antigen-specific IgG1. IgG1 is the predominant antibody isotype in the mouse serum and further induced during the response to an antigen that elicits Th2 responses. Therefore, B cell deficient (B cell KO) and matching wild type mice were sensitized and then challenged with a Th2 antigen and PM_2.5_. Right ventricular systolic pressure, type and severity of inflammation in the lungs, severe thickening of pulmonary arteries, and the molecular response in the right ventricle were measured.

## Materials and Methods

### Ethics Statement

All animal experiments were performed according to guidelines outlined by the United States Department of Agriculture and the American Association of Laboratory Animal Care under the supervision and specific approval of the Institutional Animal Care and Use Committee at New York University Langone Medical Center, New York, NY. The protocol was approved by the Institutional Animal Care and Use Committee at New York University Medical Center, New York, NY, the specific protocol number was: #111107. All efforts were made to provide mouse-appropriate housing, avoid stress, pain or suffering of the mice.

### Experimental Procedures

#### Mice

C57BL/6 wild type (000664) and B cell KO mice (muMT, C57BL/6 strain, 002288) were from Jackson Laboratory. Female mice were used for the study being 5–7 weeks of age at the start of the experiment. This is important aspect of the animal model because women are over-proportionally affected by pulmonary hypertension. For each experiment, littermate mice were randomized into cages holding up to 4 mice each. Wild type animals (originally purchased from Jackson Labs were bred in our facility in the same room as the B cell KO mice. The mice were housed under specific pathogen free conditions at the Department of Environmental Medicine, NYU Medical Center, Tuxedo, NY.

#### Urban ambient particulate matter (PM_2.5_)

The urban particulate matter was captured from a roof of a low-rise building in New York City [40]. A large batch of PM_2.5_ from the New York ambient air has been prepared and is available to all interested investigators upon request. This is size fractionated and only the PM_2.5_ fraction was used. The PM_2.5_ was resuspended and ultrasonicated before use [40]. A large batch of PM_2.5_ from the New York ambient air has been prepared and is available to all interested investigators upon request. PM_2.5_ was given intranasally at a dose of 25 μg / 50 μl in phosphate buffered saline (PBS) combined with Ovalbumin (OVA, see below). When given without OVA, the PM_2.5_ at the 25 μg / 50 μl dose did not elicit significant airway inflammation or vascular remodeling [5].

#### Sensitization and challenge with antigen and PM_2.5_

**Fig 1:**
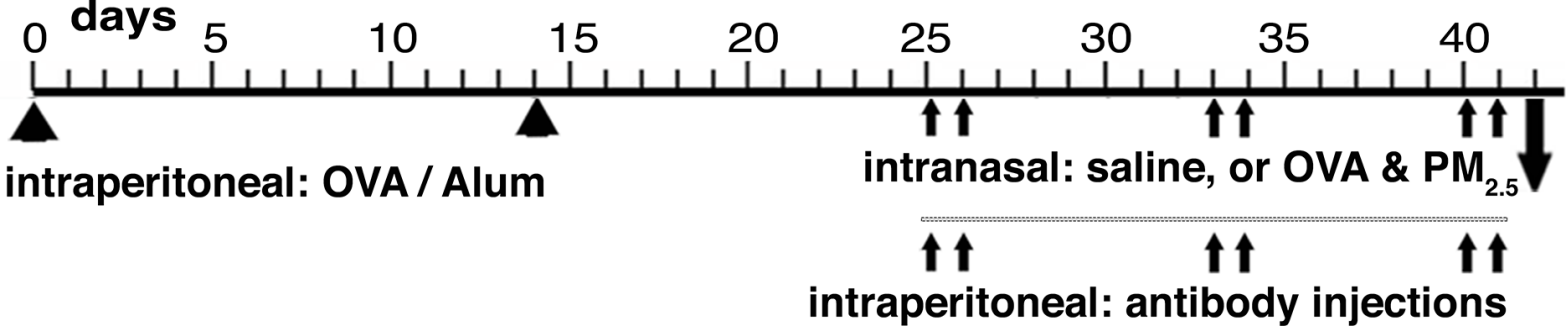
Experimental Design. Schematic representation of the timing of sensitization by intraperitoneal injection of OVA-Alum; intranasal administration of saline or antigen and PM_2.5_ (OVA & PM_2.5_); and the antibody injections administered intraperitoneally.

Using the schedule shown in Fig 1, sensitization with Ovalbumin (OVA) complexed to Alum was followed by intranasal administration of saline, or OVA (100 μg/dose) combined with PM_2.5_ (25 μg/dose) in 50 μl saline in mice anesthetized with inhaled isofluorane [16, 41].

#### Antibody Injections

B cell KO mice were injected intraperitoneally with monoclonal mouse-anti-OVA (Sigma clone OVA-14), or mouse IgG1 isotype antibody (BioXCell) at 400 μg/dose prior to the intranasal challenges. All antibodies were either purchased as low-endotoxin grade, or subjected to endotoxin removal (Endo Trap red 1/1 Chromatography endotoxin removal system, Hyglos, 82347 Bernried, Germany) prior to use.

#### Right Ventricular Systolic Pressure (RVSP)

RVSP was measured following inserting a catheter via the jugular vein in mice anaesthetized with Avertin (made by mixing 5 ml of 2-Methyl-2-butanol and 5 g of 2,2,2-Tribromoethanol; 0.25 ml of the stock solution was diluted with 10 ml of saline solution; the mice were injected according to their weight, 10 μl / g mouse) in spontaneously breathing mice [16, 41, 42]. Mice were analyzed without prior knowledge of group identity. During the experiments, we alternated mice from different cages to eliminate ‘cage effects’. The data from three independent experiments were pooled.

#### Further analysis of the animals

Following right ventricular pressure measurements, mice were euthanized with barbiturate (400 μl of 2.6% barbiturate solution) and tissues were harvested for further analysis. We did not get the full data set (RVSP, lymph node, lung gene expression, lung histology, right ventricle weight, right ventricle gene expression) in every single mouse. This is the reason for the slight differences in mouse number (n) per group between the different experiments.

**Right Ventricular Hypertrophy** [42] was measured as right ventricular (RV) weight relative to the weight of the left ventricle and septum (LV+S) or as right ventricular (RV) weight relative to body weight (BW).

**Bronchoalveolar lavage (BAL) and tissue harvest** were performed following right heart catheterization and euthanasia of the animals [15, 41, 42]. BAL was performed by gently washing with three 1 ml aliquot of Hanks Balanced Salt solutions. Following BAL, lungs, lymph nodes and ventricles were recovered. The right lung lobe was snap frozen in liquid nitrogen, the remainder of the lungs was inflated with buffered formaldehyde and removed into formaldehyde for histology. In our laboratory, the BAL does not wash out all inflammatory cells from the lungs, and is performed gently to minimize the potential for mechanical damage of the tissue. This consecutive protocol allows us to reduce the number of animals used for each study. All measurements were performed without prior knowledge of group designation of the mice.

**Severe arterial thickening** was determined on lung sections stained with hematoxylin and eosin. The severe arterial thickening change induced by prolonged exposure to antigen has been described in detail by our group [15]. The number of arteries (blood vessels adjacent to airways) at 200x magnification that showed severely thickened walls characterized by disorganized layers of cells (cells in the blood vessel wall assume a pattern that differs from the lumen) [15] were counted relative to all arteries scored per lung. All arteries with a diameter of < 100μm from at least 20 consecutive view fields comprised the sum of arteries with normal (thin) wall, mildly thickened wall, or the above-mentioned severely thickened wall. For each lung severe arterial thickening was calculated by the following formula: 100 ÷ number of all arteries examined × number of severely remodeled arteries. The muscularized nature of the severe remodeling [15] was confirmed by immunohistochemistry with anti-smooth muscle acting staining. Digital photomicrographs were obtained from images captured by scanning the hematoxylin-eosin stained lung slides using the Leica SCN400F whole slide scanning system at the Histopathology Core of NYU Medical Center and capturing images via SlidePath's Digital Image Hub (Leica Biosystems, Buffalo Grove, IL). All measurements were performed without prior knowledge of the group designation of the lungs.

#### Inflammation Scores

Hematoxylin-Eosin stained lung sections were scored for airway and interstitial (alveolar) inflammation as published [15, 41, 43, 44]: airway (peribronchial, perivascular) inflammation was scored on 20 or more consecutive view fields: 1, normal with very few inflammatory cells; 2, scattered inflammatory cells up to two rings in depth; and 3, cuffs of inflammatory cells measuring three rings or more in depth. Interstitial (alveolar) inflammation was scored on 20 or more consecutive view fields: 1, normal; 2, increased numbers of cells within the alveoli; and 3, consistent increase in the numbers of cells within the alveoli, appearance of multinucleated giant cells, and thickening of the alveolar septa. All measurements were performed without prior knowledge of the group designation of the lungs. Digital photomicrographs were obtained from images captured by scanning the hematoxylin-eosin stained lung slides using the Leica SCN400F whole slide scanning system at the Histopathology Core of NYU Medical Center and capturing images via SlidePath's Digital Image Hub (Leica Biosystems, Buffalo Grove, IL).

**Gene expression** was performed as described [16, 41]. Total RNA from lung or right heart tissue was isolated with the RNeasy Mini Kit (QIAGEN Inc, Valencia CA). Reverse transcription was performed using the High-Capacity cDNA Reverse Transcription kit for mRNA (Applied Biosystems / Life Technologies, Grand Island, NY) or Universal RT cDNA synthesis kit (Exiqon, Woburn, MA) for microRNA (miRNA). Q-PCR was performed in duplicate with 20 ng of cDNA for mRNA; or 0.1ng of cDNA for miRNA using the 7900HT Fast Real-Time PCR system (Applied Biosystems). The qPCR for the detection of mRNA expression was performed with SYBR Green (Invitrogen, Grand Island, NY). For the specific detection of the homologous genes *resistin like molecule (RELM)α*, *RELMβ*, *RELMγ* the TaqMan Gene expression Assay (Applied Biosystems) was used with FAM labeled probes and the corresponding TaqMan gene expression assay for *β-actin*. The sequences for the primers or probes, respectively, are indicated in Table 1. The following conditions were used: 95°C for 10 min, followed by 45 cycles of 95°C for 15 s and 60°C for 1 min, followed by a hold at 4°C. The microRNA (*miR)-135a* expression level was determined by using *miR-135a* with LNA-modified primers (Exiqon) and SYBR Green master mix (Exiqon) with these conditions: 95°C for 10 min, followed by 45 cycles of 95°C for 10 s and 60°C for 1 min, followed by a hold at 4°C.

**Table 1:** Sequences of primers and probes.

Name	Gene name abbreviation	Sequence
IL-6-F	*Il6*	TACCACTTCACAAGTCGGAGGC
IL-6-R	*Il6*	CTGCAAGTGCATCATCGTTGTTC
IL-13-F	*Il13*	AACGGCAGCATGGTATGGAGTG
IL-13-R	*Il13*	TGGGTCCTGTAGATGGCATTGC
IL-17A-F	*Il17a*	CAGACTACCTCAACCGTTCCAC
IL-17A-R	*Il17a*	TCCAGCTTTCCCTCCGCATTGA
IL-17F-F	*Il17f*	AACCAGGGCATTTCTGTCCCAC
IL-17F-R	*Il17f*	GGCATTGATGCAGCCTGAGTGT
IL-33-F	*Il33*	ACTGCATGAGACTCCGTTCTG
IL-33-R	*Il33*	CCTAGAATCCCGTGGATAGGC
MMP12-F	*Mmp12*	CACACTTCCCAGGAATCAAGCC
MMP12-R	*Mmp12*	TTTGGTGACACGACGGAACAGG
BNP-F	*Nppb*	TCCTAGCCAGTCTCCAGAGCAA
BNP-R	*Nppb*	GGTCCTTCAAGAGCTGTCTCTG
S100a8-F	*S100a8*	CAAGGAAATCACCATGCCCTCTA
S100a8-R	*S100a8*	ACCATCGCAAGGAACTCCTCGA
S100a9-F	*S100a9*	TGGTGGAAGCACAGTTGGCAAC
S100a9-R	*S100a9*	CAGCATCATACACTCCTCAAAGC
β-actin-F	*Actb*	GGCTGTATTCCCCTCCATCG
β-actin-R	*Actb*	CCAGTTGGTAACAATGCCATGT
RELMα (TaqMan)	*Retnla*	CTTGCCAATCCAGCTAACTATCCCT
RELMβ (TaqMan)	*Retnlb*	GGAAGCTCTCAGTCGTCAAGAGCCT
RELMγ (TaqMan)	*Retnlg*	AAACCTGGCTCATATCCCATTGATG
Actin, β (TaqMan)	*Actb*	ACTGAGCTGCGTTTTACACCCTTTC
miR-135a (Exiqon)	*miR-135a*	UAUGGCUUUUUAUUCCUAUGUGA

Abbreviations: F- forward, R-reverse

Raw data were analyzed with SDS Relative Quantification Software version 2.3 (Applied Biosystems) to determine cycle threshold (Ct). Mean Ct values were standardized by calculating ΔCt using the housekeeping genes β-actin (mRNA), or 5S (miRNA) and calculating 1.98^ΔCt^ ×10,000.

Gene expression is indicated as fold-increase over the mean of the wild type saline, or the wild type OVA-PM_2._5, or the B cell KO saline groups.

#### Immune responses

BAL cells and lymph node cells were analyzed by flow cytometry as described [15, 43, 44] using a MACS Quant (Miltenyi Biotec, Auburn, CA) instrument and FloJo (TreeStar Inc, Ashland, OR) software. Cell counts were performed using the feature provided by the MACS Quant flow cytometer. All cell analysis was performed without prior knowledge of the group designation of the samples. BAL samples were analyzed [41, 43] for the numbers of eosinophils (CD11b^high^, CCR3^high^, GR1^low^, CD11c^low-intermediate^, major histocompatibility complex class II (MHCII)^low-intermediate^), neutrophils (CD11b^high^, GR1^high^, CD11c^low-intermediate^, MHCII^low-intermediate^), and CD11c+ cells. The mean fluorescence intensity of CD11c+ cells stained with anti MHCII antibody was determined as a measure of the capacity of these airway dendritic cells / airway macrophages to present antigen [43, 44].

#### Lymph Node Cells

Single cell suspensions from lung draining lymph nodes were analyzed as described [15, 43–45] following brief fixation with 2% PBS buffered formaldehyde (20–30 minutes at room temperature). The cell suspensions were analyzed for the numbers of T cells (CD3 positive, B220 negative cells that are also positive for CD4).

Intracellular cytokine staining was performed on cell suspensions prepared from the lung draining lymph nodes, as previously described [15, 43–46]. The cells were cultured in the presence of phorbol-myristate-acetate (PMA) and ionomycin for 4 h, with the addition of Brefeldin A for the last 2 h of culture. The cells were harvested, fixed in 2% buffered formaldehyde, permeabilized, and stained with anti-cytokine (anti-IL-13, anti–IFNγ and anti- IL-17A/F monoclonal antibodies). The cells were surface stained with anti-CD3 combined with anti-CD8α and anti-CD4 labeled monoclonal antibodies. The cells were examined on a MACSQuant cytometer. Electronic gates were set using the forward and side scatter profiles in combination with the surface labels to capture CD3+ T cells that were also CD4+. Intracellular isotype control monoclonal antibodies were used to set the quadrants that demarcated cytokine-positive cells.

The monoclonal antibodies for flow cytometry were tagged with Pacific Blue, AlexaFluor, fluorescein (FITC), phycoerythrin (PE), peridinin-chlorophyll (PerCP), Allophycocyanin (APC), or cyanine (Cy) tandem dyes that were purchased from BD Bioscience (San Jose, California), Ebioscience (San Diego, California) or Biolegend (San Diego, California), the following clones were used: anti-CD11c-PacificBlue (clone N418); anti-CD11b- PE (clone M1/70); anti-MHCII-FITC (I-A/I-E, clone M5/114.15.2); anti-Ly-6G/Ly-6C-APC-Cy7 (clone GR1); anti-CCR3-AlexaFluor 647 (clone TG14, Biolegend, or clone 83103, BD Bioscience); PerCp, or APC-Cy7-anti-CD4 (clone GK1.5); anti-CD3-FITC (clone 17A2, ebioscience); anti-IL-13-PE (clone eBio13A, Ebioscience); anti-IL-17A/F-PE-Cy7 (clone TC11-18H10.1, Biolegend). IgG1-isotype control antibodies used to set the gates were from ebioscience or biolegend.

#### OVA specific antibody titers

The ELISA was performed as described [41, 44] with slight modifications. A standard serum pool was created from 22 sensitized and OVA-PM challenged wild type mice. The plates were developed with Super Aqua Blue substrate (ebioscience), analyzed with SoftMax Pro software, and for each sample and well the calculated concentration value was multiplied by the dilution factor. All serum samples were assayed in one experiment.

#### Statistical analysis

Statistical analysis was performed with the Aable 3 (Gigawiz) or Prism 6 (Graphpad) software. Data sets were analyzed for correlation using the Spearman’s Rank Correlation test. Contingency tables were analyzed with the chi-square test for multi-group comparisons and the two-tailed Fisher’s exact test for two-group comparisons. Two group comparisons were conducted with the unpaired, two-tailed Mann-Whitney U test or the unpaired, two-tailed t-test with Welsh’s correction for unequal variances. A *P* value <0.05 was considered to be significant.

## Results

### Antigen-specific IgG1 levels in wild type mice exposed to antigen and PM_2.5_

**Fig 2:**
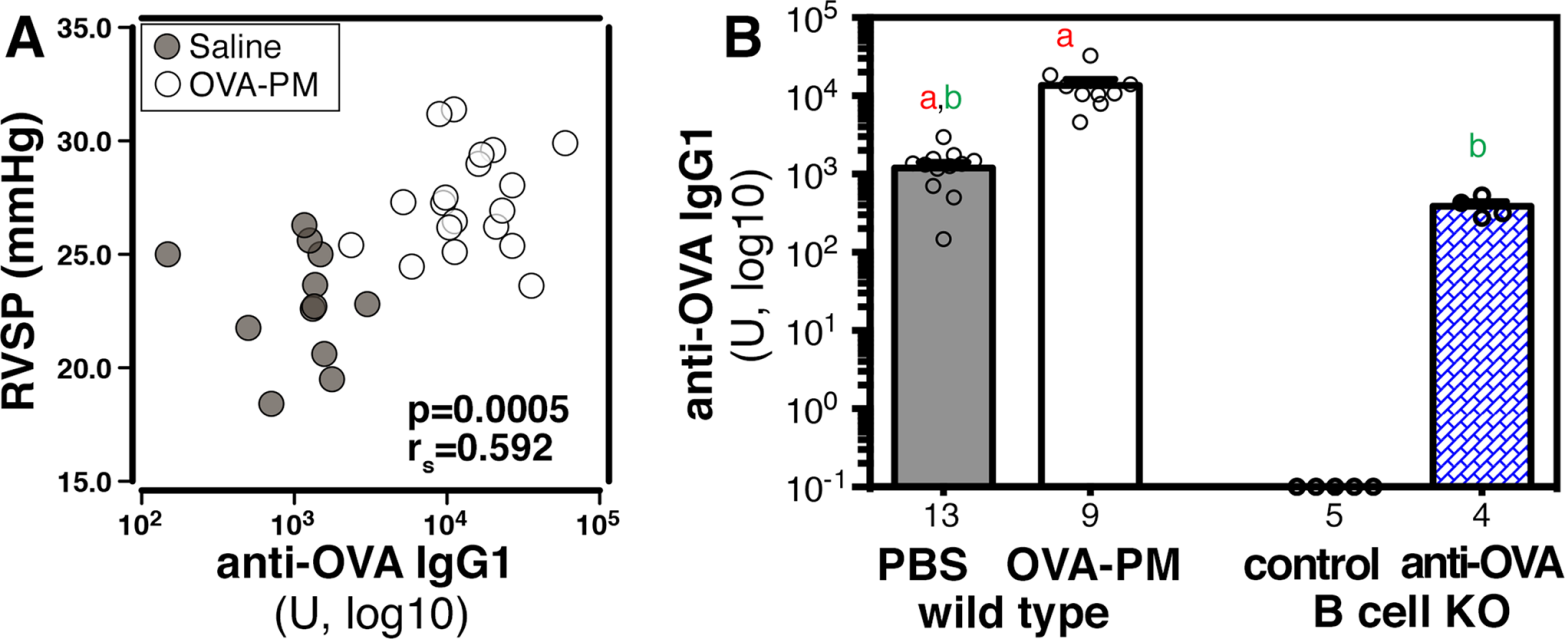
OVA-specific IgG1 serum levels in sensitized wild type mice and in B cell KO mice following reconstitution with monoclonal anti-OVA IgG1 antibody. OVA-specific IgG1 levels were measured in sera of sensitized wild type mice that were either challenged intranasally with saline or OVA-PM, and in sera of sensitized B cell KO mice that were either controls or injected with anti-OVA IgG1 antibody. (A) Correlation of IgG1 serum titers with right ventricular systolic pressures in sensitized wild type mice. Values for P and rs (tie corrected, Spearman’s rank correlation test) are indicated. (B) Bar graphs show (mean ± SEM) and individual data points of OVA-specific IgG1 levels. Pairs of letters above the bars indicate the pairs of groups that showed significant differences (p<0.05) calculated with the t-test with Welch’s correction (unpaired, two-tailed). Numbers below the bars indicate the numbers of mice per group.

The experimental schedule shown in Fig 1 was used to ask if right ventricular systolic pressure would be correlated with antigen specific IgG1, the major isotype produced during Th2 responses in mice. Serum antigen-specific IgG1 was highly significantly correlated with right ventricular systolic pressure (Fig 2A) and antigen-specific IgG1 titers were, as expected, significantly further increased in sensitized wild type mice upon challenge with OVA-PM_2.5_ (Fig 2B). Furthermore, co-neutralizing IL-13 and IL-17A resulted in a significant amelioration of the mean increase in antigen-specific IgG1 in sensitized mice exposed to the antigen and PM_2.5_ (18,170 ± 2,819 U in OVA-PM challenged control mice vs. 9,321 ± 2,146 U in OVA-PM challenged mice given neutralizing anti-IL-13 and anti-IL-17A antibodies, p = 0.013 Mann-Whitney U test).

### B cells are necessary for the increase in right ventricular systolic pressure

The correlation between the IgG1 responses and right ventricular systolic pressures could be a reflection of the strength of the immune response induced by antigen and PM_2.5_ challenge. In that case, the antigen-specific IgG1 would be a marker of the preferentially induced antibody isotype by the priming protocol [47]. Alternatively, the highly significant correlation between the antigen-specific IgG1 antibody concentrations and right ventricular systolic pressure could indicate a mechanistic relation. We studied B cell KO mice next to distinguish between these two possibilities.

**Fig 3:**
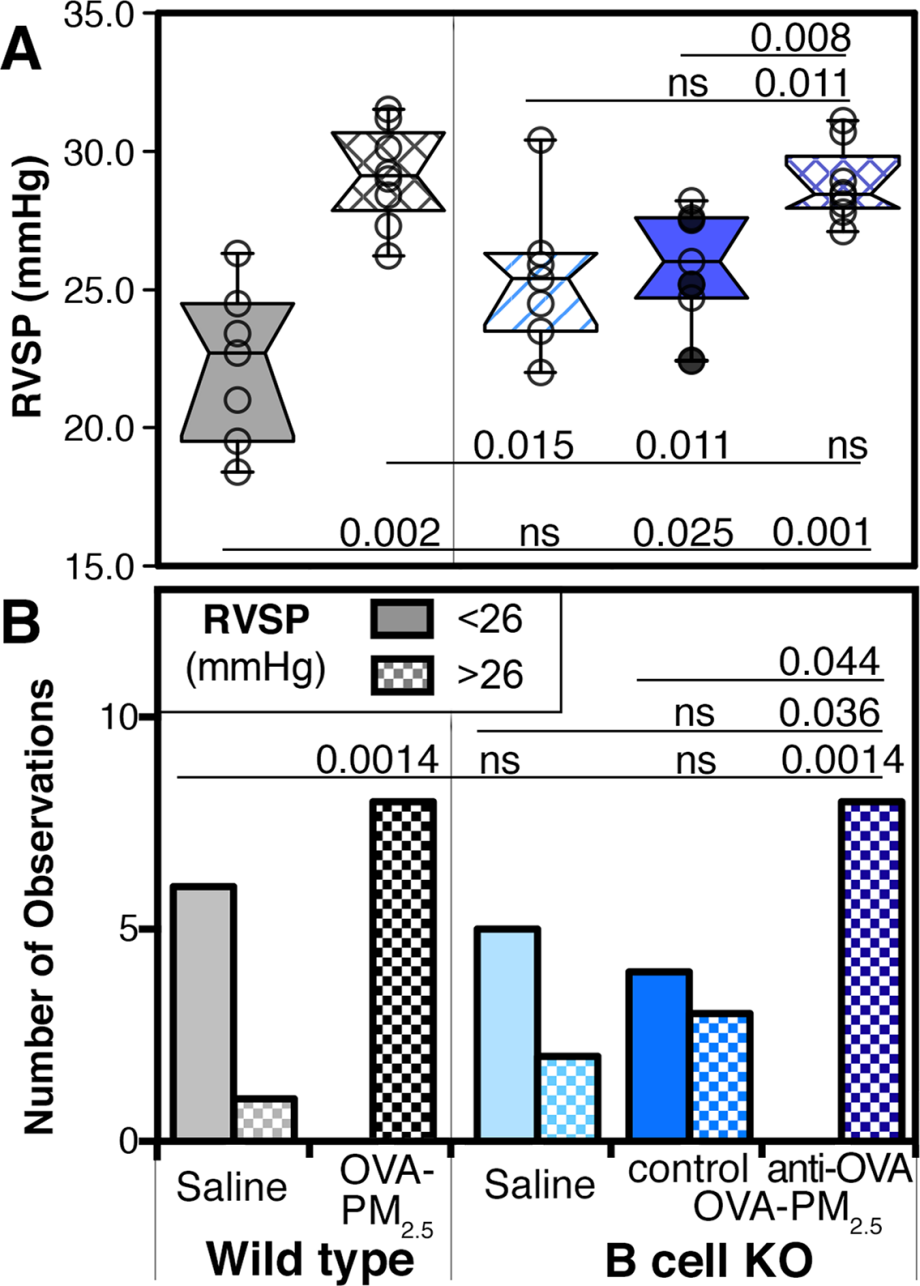
Right ventricular systolic pressures in wild type and B cell KO mice. Wild type and B cell KO mice were challenged with saline, or antigen and PM_2.5_ (OVA-PM_2.5_) intranasally. Data were pooled from 3 experiments; circles represent the data from individual mice, n = 7–8 per group. OVA-PM_2.5_ challenged B cell KO control mice were injected with control antibody (open circles) or given no injections (filled circles). Another group of B cell KO mice was injected with antigen specific IgG1 (anti-OVA monoclonal). Right ventricular systolic pressure (RVSP, mmHg) data are shown as box plot of the medians **(A)** or as bar graph of the numbers of mice with RVSP less or greater than 26 mmHg **(B)**. The left-most beginning of each horizontal line indicates the group with which pairwise comparisons were made using the Mann-Whitney U test **(A)** or Fisher’s exact test **(B)**. Significant P values (P<0.05) are indicated; ns: not significant.

Groups of B cell KO and wild type mice (Fig 3A) were given the sensitization and challenge protocol shown in Fig 2. We measured right ventricular systolic pressure via a catheter inserted through the jugular vein using a cutting edge technique in anesthetized, spontaneously breathing mice [42]. In mice, right ventricular systolic pressure is a surrogate because the animals are too small for direct pulmonary artery pressure measurements. We compared group medians (Fig 3B), and calculated the number of animals that had right ventricular systolic pressure measurements below or above a 26 mmHg threshold (Fig 3C). Our data showed that sensitized wild type mice had the expected robust and significant increase in right ventricular systolic pressure (p = 0.002) following challenge with antigen and PM_2.5_ [5, 16]. In contrast, similarly treated B cell KO mice failed to do so (Fig 3B and 3C).

### Administration of antigen-specific IgG1 restored right ventricular systolic pressure responses in B cell KO mice exposed to the antigen and PM_2.5_

To identify the role of antigen-specific IgG1, we injected sensitized B cell KO mice with mouse monoclonal anti-Ovalbumin (OVA) IgG1 during the intranasal challenge period. At the end of the experiment, the antigen-specific IgG1 antibodies were measured. Reconstituted B cell KO mice had an IgG1 serum concentration within the range of OVA-specific IgG1 measured in sera of saline exposed, sensitized wild type mice (Fig 2B).

Our data showed that B cell KO mice injected with the antigen-specific IgG1 during the intranasal challenge period developed significantly increased right ventricular systolic pressure (Fig 3). The significance levels were 0.011 for comparison to saline exposed B cell KO mice, 0.008 for comparison to OVA-PM_2.5_ control B cell KO mice, >0.05-not-significant for comparison to OVA-PM_2.5_ exposed wild type mice (Fig 3). The OVA-PM_2.5_ B cell KO control group consisted of mice that received no antibody injections (Fig 3B; black filled circles), and B cell KO mice that were injected with control IgG1 (Fig 3B; open circles).

### Similar IL-13 and IL-17A production in wild type and B cell KO mice following challenge with antigen and PM_2.5_

Antigen-specific IgG1 injections could have changed the T cell response, increased cytokine production, or changed the inflammatory response. These possibilities could account for the increased right ventricular systolic pressure following antigen-specific IgG1 injection in B cell KO mice challenged with the antigen and PM2.5. Our previous data [16] had shown that the increased right ventricular systolic pressure induced by exposure with antigen and PM_2.5_ in wild type mice was significantly ameliorated by co-neutralizing IL-13 and IL-17A. Thus, our attention focused on these two cytokines in the B cell KO mice.

**Fig 4:**
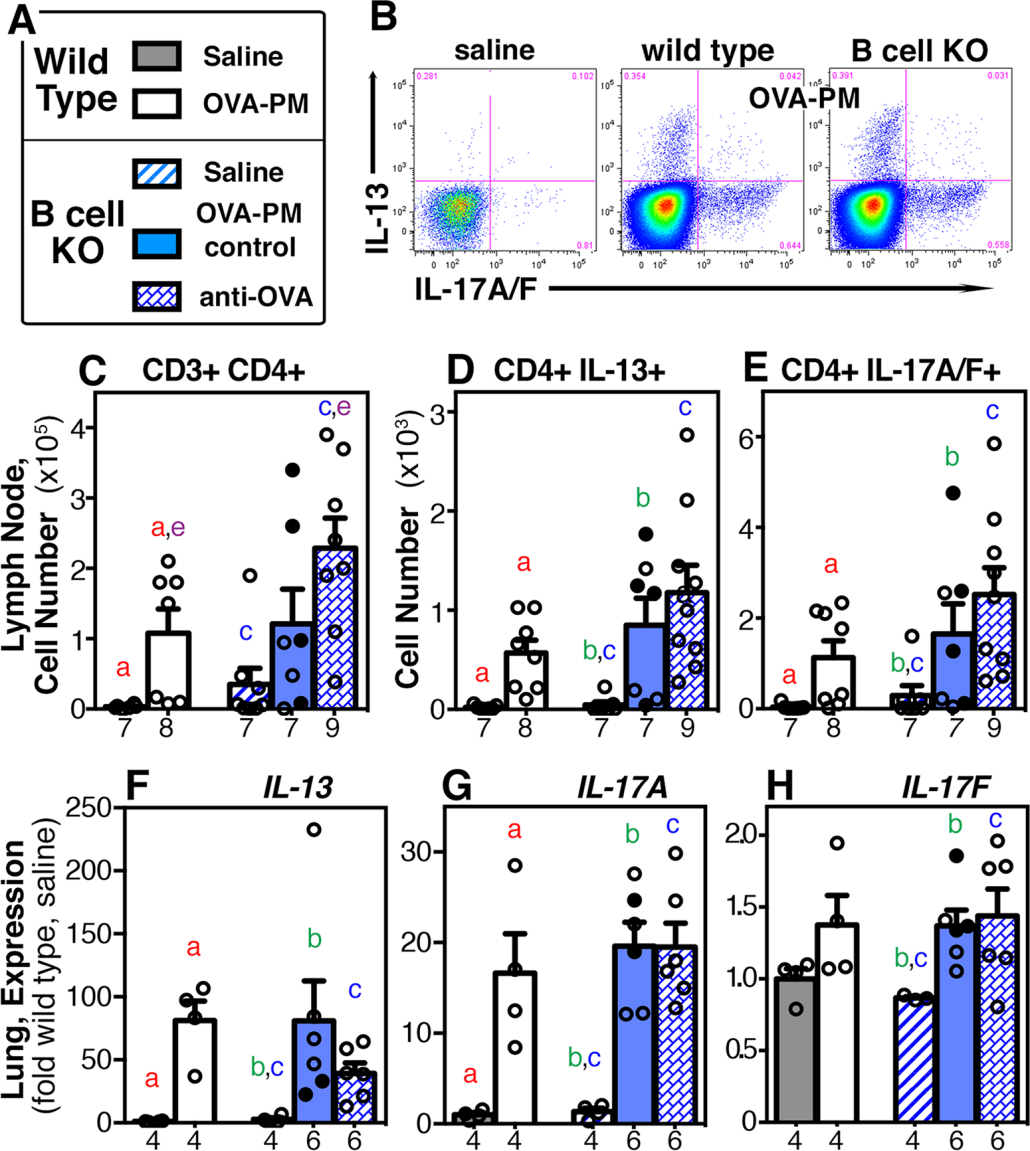
IL-13 & IL-17A response to exposure with OVA-PM_2.5_ in wild type and B cell KO mice. Lymph node and lung tissues from groups of wild type or B cell KO mice challenged with saline or OVA-PM were analyzed. Groups of OVA-PM challenged B cell KO mice were either controls [injected with control antibody (open circles) or given no injections (filled circles)] or injected with anti-OVA IgG1 antibody. **(A)** Legend. **(B)** Representative dot plots were generated by flow cytometry of CD4+ T cells (CD3-CD4-dual-positive) showing staining for IL-13 vs. IL-17A. **(C-E)** The flow cytometry data were numerically analyzed to calculate the numbers for each cell type (mean ± SEM) per lung draining lymph node. **(F-H)** Gene expression in the lungs of IL-13, IL-17A, IL-17F is indicated (mean ± SEM) as fold-increase over the means of the wild type saline group. Pairs of letters above the bars indicate the pairs of groups that showed significant differences (p<0.05) calculated with the Mann Whitney U test or the t-test with Welch’s correction (unpaired, two-tailed tests). Numbers below the bars indicate the numbers of mice per group.

Lung draining lymph node cells were examined by flow cytometry and lung tissue for gene expression (Fig 4). All antigen and PM_2.5_ challenged wild type and B cell KO groups showed similar, significant increases in the numbers of IL-13-positive and IL-17A-positive T cells in the lymph nodes, and in gene expression of *IL-13*, *IL-17A* and *IL-17F* in the lungs (Fig 4). However, antigen and PM_2.5_ challenged B cell KO mice injected with antigen-specific IgG1 had significantly more CD4 T cells in the lung draining lymph nodes when compared to antigen and PM_2.5_ challenged wild type mice (Fig 4C).

### Significantly different inflammation phenotype in B cell KO mice challenged with antigen and PM_2.5_

**Fig 5:**
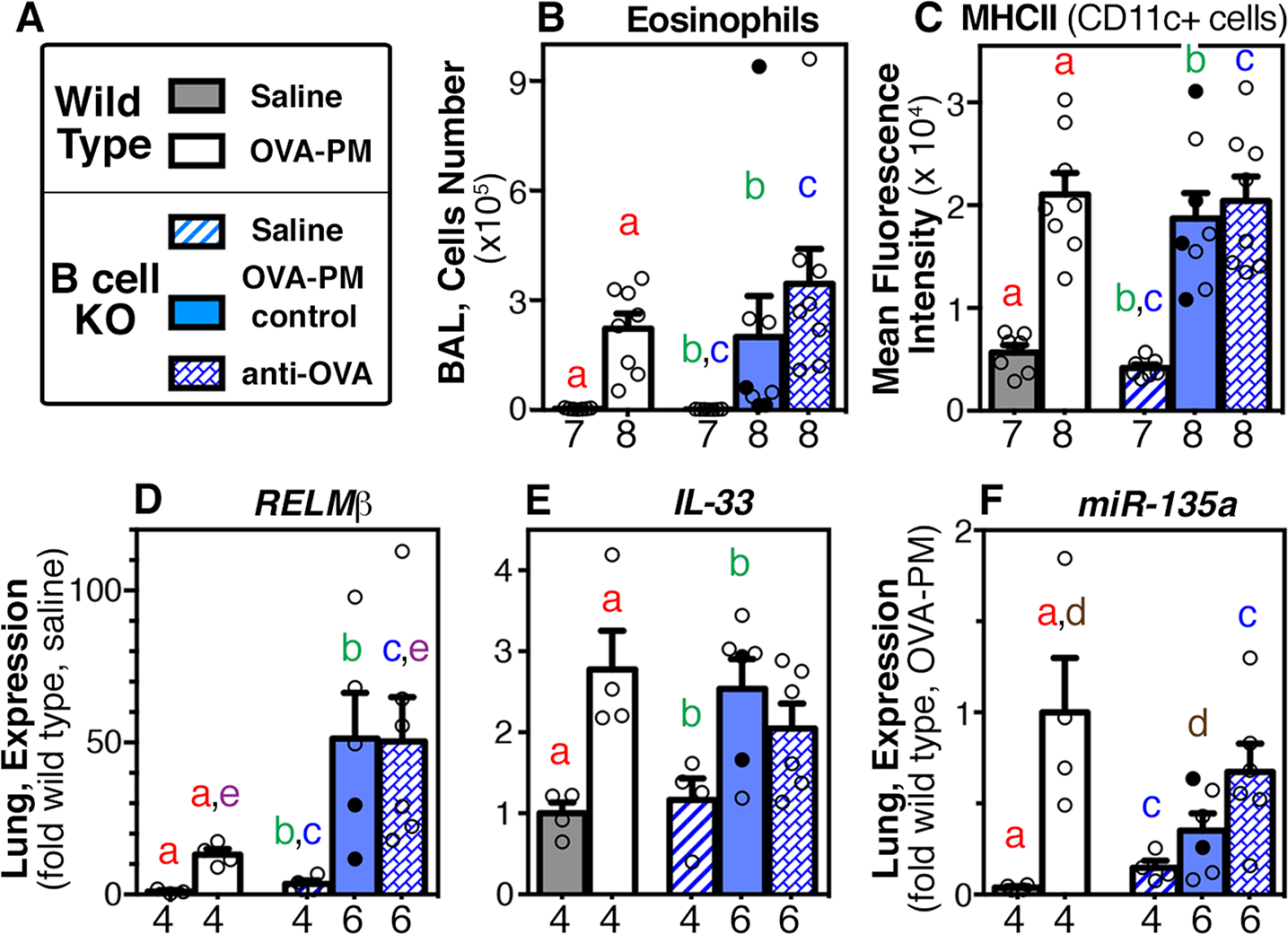
Markers of T (helper)h2 inflammation in the lungs. Groups of wild type or B cell KO mice challenged with saline or OVA-PM were analyzed. Groups of OVA-PM challenged B cell KO mice were either controls [injected with control antibody (open circles) or given no injections (filled circles)] or injected with anti-OVA IgG1 antibody. **(A)** Legend. **(B-F)** Bar graphs show mean ± SEM and individual data points for **(B)** numbers of bronchoalveolar lavage (BAL) eosinophils; **(C)** mean fluorescent intensity (MFI) of major histocompatibility complex class II (MHCII) of BAL CD11c+ cells; **(D)** RELMβ; **(E)** IL-33; and **(F)** miR-135a expression in the lungs. Gene expression in the lungs is shown as fold-increase over the means of the wild type saline group. Pairs of letters above the bars indicate the pairs of groups that showed significant differences (p<0.05) calculated with the Mann Whitney U test or the t-test with Welch’s correction (unpaired, two-tailed tests). Numbers below the bars indicate the numbers of mice per group.

**Fig 6:**
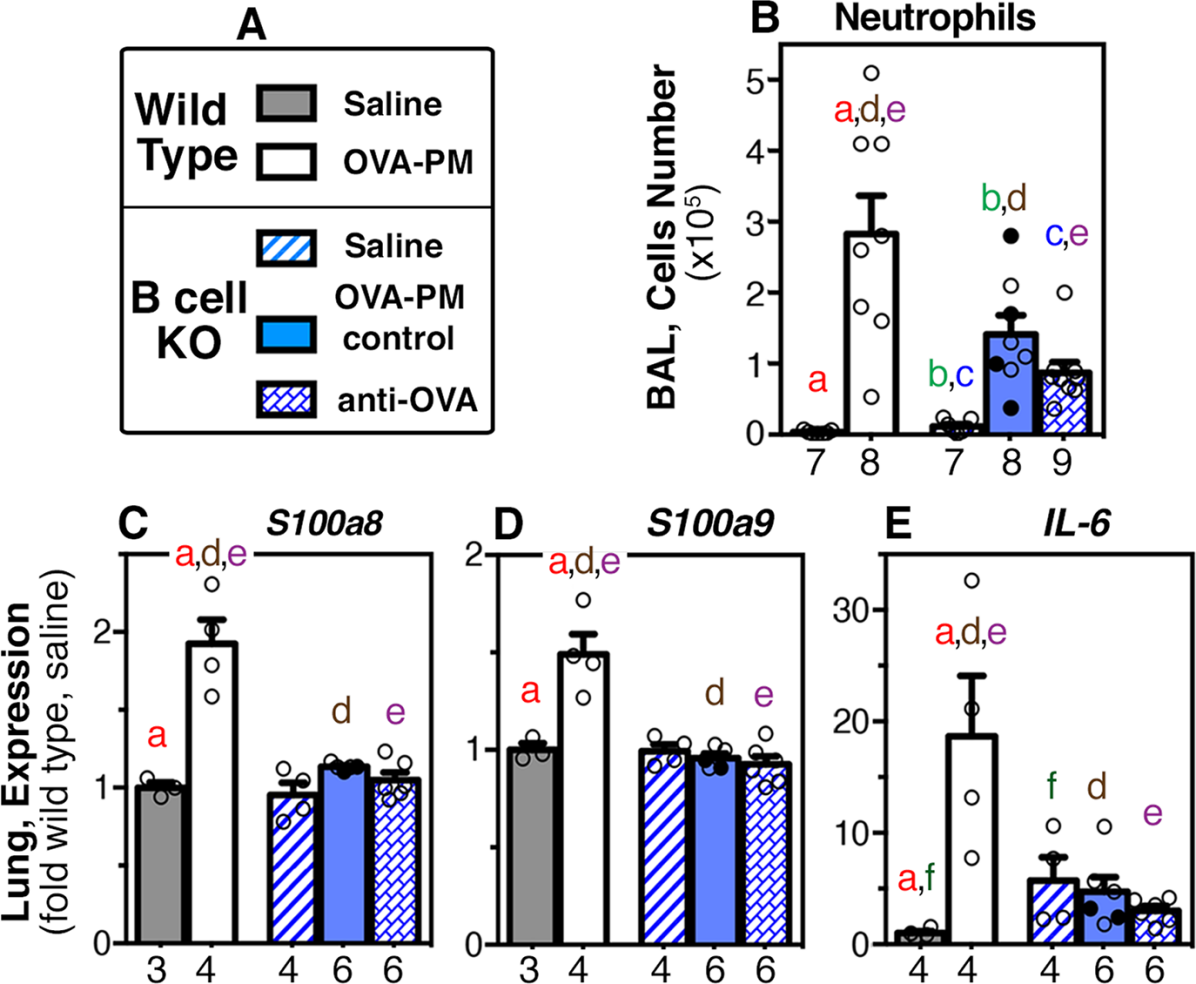
Markers of Th17 inflammation in the lungs. Groups of wild type or B cell KO mice challenged with saline or OVA-PM were analyzed. Groups of OVA-PM challenged B cell KO mice were either controls [injected with control antibody (open circles) or given no injections (filled circles)] or injected with anti-OVA IgG1 antibody. **(A)** Legend. **(B-E)** Bar graphs show mean ± SEM and individual data points for **(B)** numbers of BAL neutrophils; **(C)** S100a8; **(D)** S100a9; and **(E)** IL-6 gene expression. Gene expression in the lungs is shown as fold-increase over the means of the wild type saline group. Pairs of letters above the bars indicate the pairs of groups that showed significant differences (p<0.05) calculated with the Mann Whitney U test or the t-test with Welch’s correction (unpaired, two-tailed tests). Numbers below the bars indicate the numbers of mice per group.

**Fig 7:**
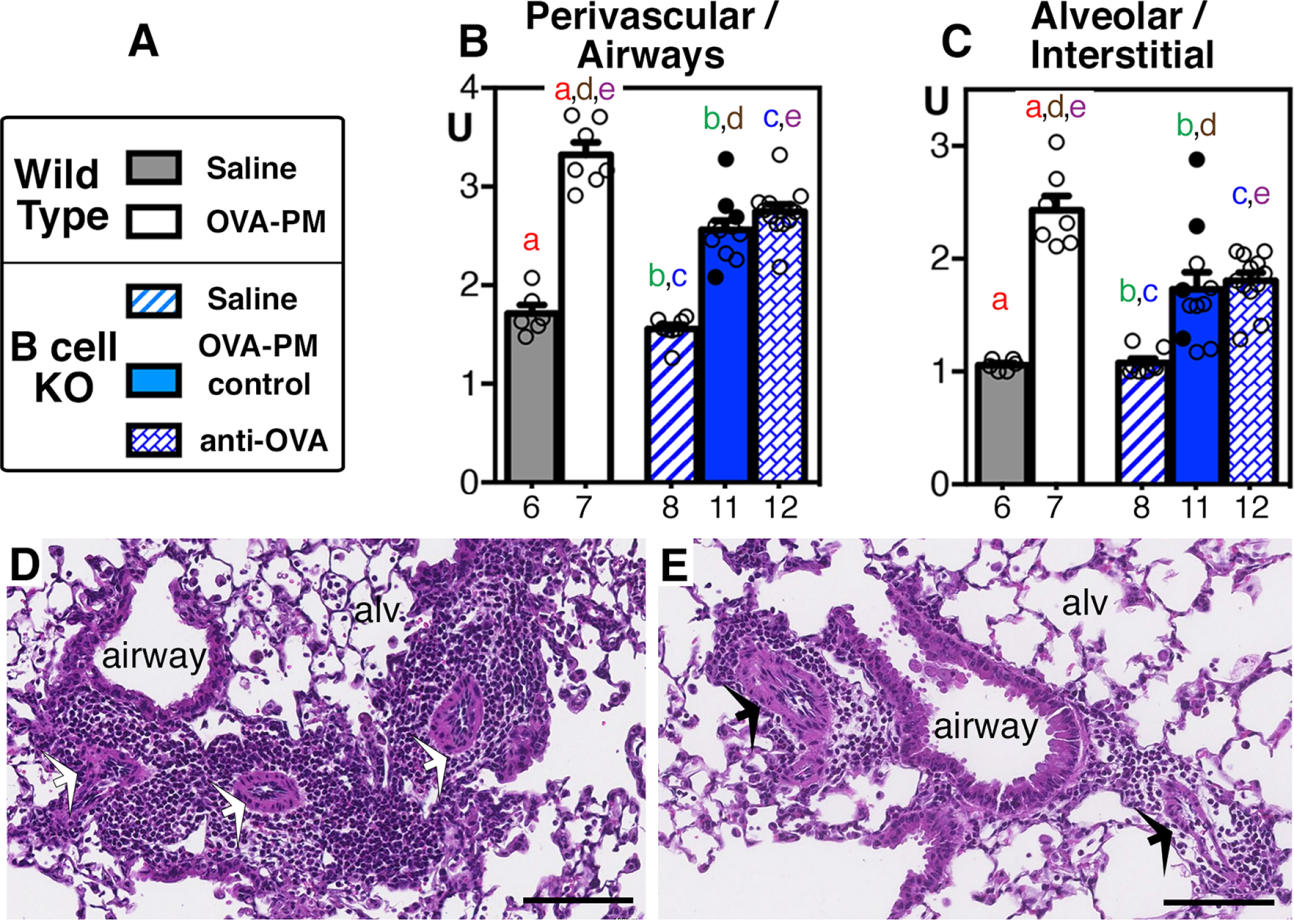
Inflammatory cell infiltrates in the lungs. Groups of wild type or B cell KO mice challenged with saline or OVA-PM were analyzed. Groups of OVA-PM challenged B cell KO mice were either controls [injected with control antibody (open circles) or given no injections (filled circles)] or injected with anti-OVA IgG1 antibody. **(A)** Legend. **(B,C)** Bar graphs show mean ± SEM and individual data for inflammatory cell infiltrates (scores, U) accumulating **(B)** perivascularly / peribronchially or in the **(C)** alveoli / interstitium of the lungs. Pairs of letters above the bars indicate the pairs of groups that showed significant differences (p<0.05) calculated with the Mann Whitney U test (unpaired, two-tailed). Numbers below the bars indicate the numbers of mice per group. **(D, E)** Photomicrographs show sections of OVA-PM2.5 exposed lungs of representative sensitized wild type **(D)** and B cell KO mice **(E**, given injections with anti-OVA IgG1). The sections were stained with hematoxylin & eosin. Scale bars indicate 100 μm; airways and alveoli (alv) are indicated; arrows point to blood vessels. Note the inflammatory infiltrates surrounding airways, blood vessels and within the alveoli.

The inflammatory markers studied in the next experiments are characterized as T helper 2 (Th2, Fig 5), Th17 (Fig 6), or tissue remodeling (Fig 7) markers and are summarized in Table 2.

**Table 2:** List of markers, their regulation, function related to tissue remodeling, and relation to T helper responses.

	Regulated by:				
Marker	IL-13	IL-17A	IL-13 & IL-17A	Function	T helper type
IL-13					Th2
IL-17A					Th17
IL-17F					Th17
BAL eosinophils					Th2
BAL: MHCII expression by CD11c+ cells	x		x	antigen presentation	
BAL neutrophils		x	x		Th17
lung RELMα	x		x	remodeling	
lung RELMβ	x		x		
lung MMP12	x		x	remodeling	
lung IL-33					Th2
lung S100a8		x	x		
lung S100a9		x	x		
lung IL-6		x	x		Th17
lung miR-135a			x		
heart RELMα				unknown	
heart RELMγ				unknown	
heart IL-33				remodeling	
heart BNP			x	molecular strain	

The numbers of BAL eosinophils were similarly highly significantly increased in all groups of wild type and B cell KO mice exposed to the antigen and PM_2.5_ (Fig 5B), as well as the IL-13-dependent markers [16], intensity of MHCII expression by CD11c+ cells in the BAL and lung expression of *RELMβ* and *IL-33* (Fig 5C–5E). All three markers were significantly increased by exposure to the antigen and PM_2.5_ as expected [16] in wild type mice and also in B cell KO mice (Fig 5C–5E). Compared with wild type animals, B cell KO mice exposed to antigen and PM_2.5_ had increased RELMβ expression in the lungs, that reached statistical significance in the group of B cell KO mice injected with antigen specific IgG1 (Fig 5D).

The inflammatory marker [48], miR-135a, was studied next because it is inhibited by co-neutralization of IL-13 and IL-17A in the lungs of wild type mice exposed with antigen and PM_2.5_ [16]. Lung expression of *miR-135a* was significantly increased in antigen and PM_2.5_ exposed wild type mice, as expected [16], and in B cell KO mice that were injected with antigen-specific IgG1 during antigen and PM_2.5_ challenge (Fig 5F). In contrast, the expression of *miR-135a* in the lungs of control B cell KO mice (challenged with antigen and PM_2.5_ with no injection of antigen-specific IgG1) was not increased when compared to saline (Fig 5F) and significantly lower when compared to wild type animals challenged with antigen and PM_2.5_ (Fig 5F).

As expected [16], the IL-17A-dependent markers were significantly increased in antigen and PM_2.5_ exposed wild type mice (Fig 6). These markers included: numbers of neutrophils in the BAL, lung expression of the danger associated molecular pattern molecules *S100 calcium binding protein A* (*S100a)8*, *S100a9* [49, 50] and lung expression of *IL-6*. IL-6 is significantly increased in the serum of pulmonary hypertension patients [51]. In contrast, in B cell KO mice, only the numbers of neutrophils in the BAL were significantly increased upon antigen and PM_2.5_ challenge but to a significantly lower level when compared to the wild type group (Fig 6B). Furthermore, *S100a8*, *S100a9* and *IL-6* expression was not increased in the lungs of B cell KO mice exposed to the antigen and PM_2.5_ when compared to saline exposed mice (Fig 6C–6E). Injection of B cell KO mice with antigen-specific IgG1 during the antigen and PM_2.5_ challenge phase failed to restore the up-regulation of IL-17A controlled markers (Fig 6). The saline control group of B cell KO mice had mildly increased *IL-6* expression in the lungs when compared to saline exposed wild type mice (Fig 6E).

The inflammation scores for histologically visible infiltrates in the lungs of antigen and PM_2.5_ challenged mice were analyzed. As expected all groups of mice challenged with antigen and PM_2.5_ had significantly increased scores when compared to saline exposed groups of mice (Fig 7). Relative to wild type mice, the scores for inflammatory cell infiltrates surrounding airways or for inflammatory cells within the alveoli and interstitium were significantly lower (p = 0.0005) in sensitized B cell KO mice exposed to antigen and PM_2.5_ irrespective of injection of antigen specific IgG1 (Fig 7).

### Severe pulmonary arterial thickening in response to the antigen and PM_2.5_ exposure is induced independently of B cells or antigen-specific IgG1

**Fig 8:**
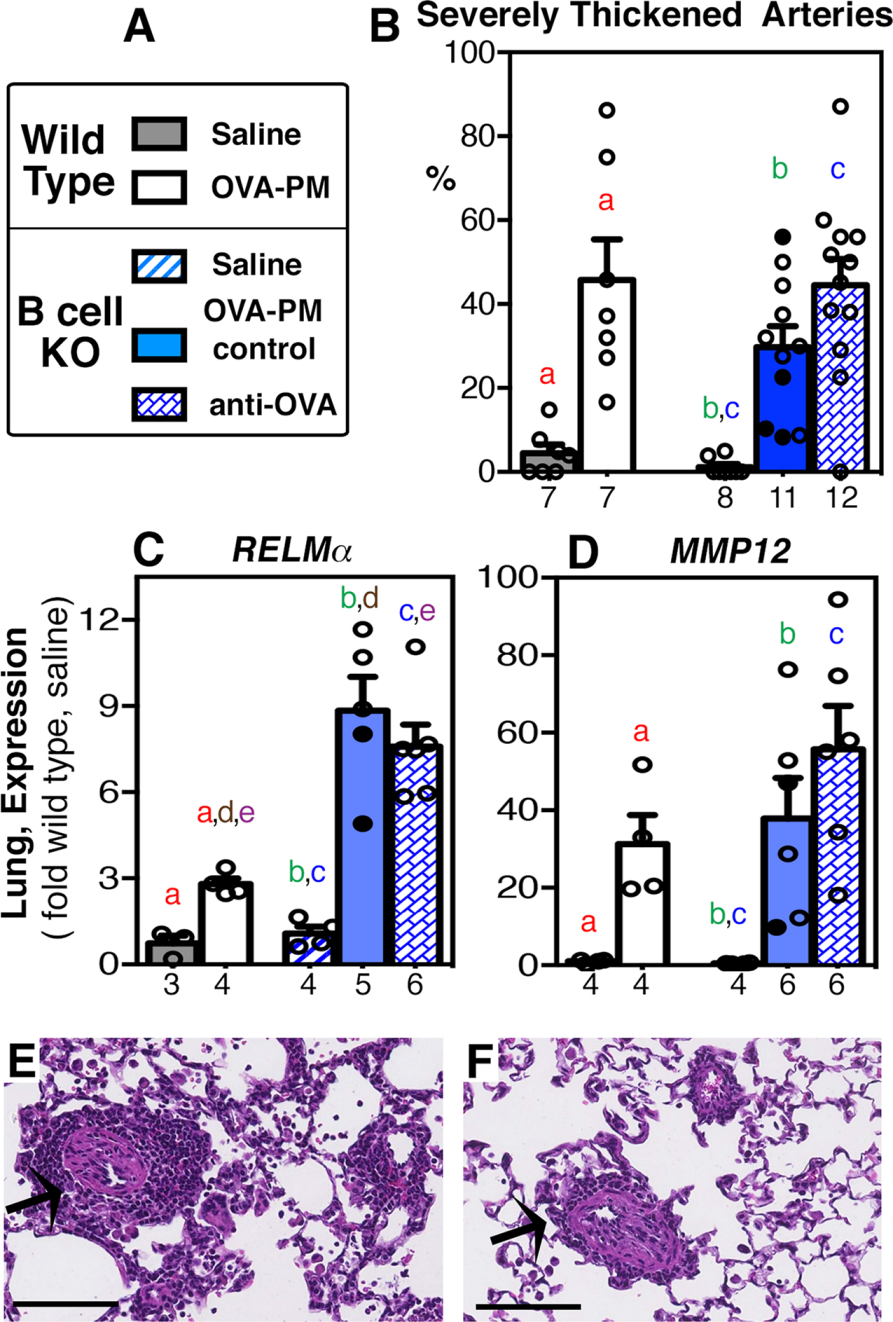
Severe thickening of pulmonary arteries and expression of pro-remodeling genes in the lungs. Groups of wild type or B cell KO mice challenged with saline or OVA-PM were analyzed. Groups of OVA-PM challenged B cell KO mice were either controls [injected with control antibody (open circles) or given no injections (filled circles)] or injected with anti-OVA IgG1 antibody. **(A)** Legend. **(B-D)** Bar graphs show mean ± SEM and individual data for **(B)** severe arterial thickening by histological analysis and for expression of pro-remodeling genes (**C** RELMα, **D** MMP12) in the lungs. Gene expression in the lungs is shown as fold-increase over the means of the wild type saline group. Pairs of letters above the bars indicate the pairs of groups that showed significant differences (p<0.05) calculated with the Mann Whitney U test or the t-test with Welch’s correction (unpaired, two-tailed tests). Numbers below the bars indicate the numbers of mice per group. **(E,F)** Photomicrographs show sections of OVA-PM2.5 exposed lungs of representative sensitized wild type **(E)** and B cell KO mice **(F**, given no injections with antibody). Sections were stained with hematoxylin & eosin. Scale bars indicate 100 μm; arrows point to severely thickened blood vessels. Note the irregular cell patterns in the remodeled vessels.

The effects of antigen-specific IgG1 could be directed against cells within the blood vessels. For example, crosslinking of FcgRIII on circulating neutrophils has been shown to promote atherosclerosis by increasing adhesion of neutrophils to endothelial cells and subsequent release of neutrophil mediators damaging the endothelial cells [52]. The morphologic correlate could be the severity of pulmonary arterial remodeling. To test this idea, the percentage of severely thickened pulmonary arteries of small-diameter (<100μm) was determined (Fig 8). The percentage of arteries that had severely thickened walls was increased from less than 10% to 50% following challenge with the antigen and PM2.5, in wild type mice and B cell KO mice injected with antigen-specific IgG1 (Fig 8B). While antigen and PM2.5 challenged control B cell KO mice had a lower mean percentage of severely thickened, small arteries (30%), this was not significantly different from the group of B cell KO mice injected with antigen-specific IgG1 (Fig 8B).

Two IL-13-dependent cytokines with pro-remodeling activity *RELMα* and *matrix-metallo-proteinase (elastase*, *MMP)12* were expressed at significantly increased levels in wild type mice (as expected [16]), and also in B cell KO mice (Fig 8C and 8D) in response to challenge with antigen and PM_2.5_ relative to the saline groups. The lung expression of *RELMα* was significantly increased in antigen and PM_2.5_ challenged B cell KO mice, when compared to similarly treated wild type mice (Fig 8C).

### Exposure to antigen and PM_2.5_ induces distinct changes in the right ventricle of B cell KO mice

**Fig 9:**
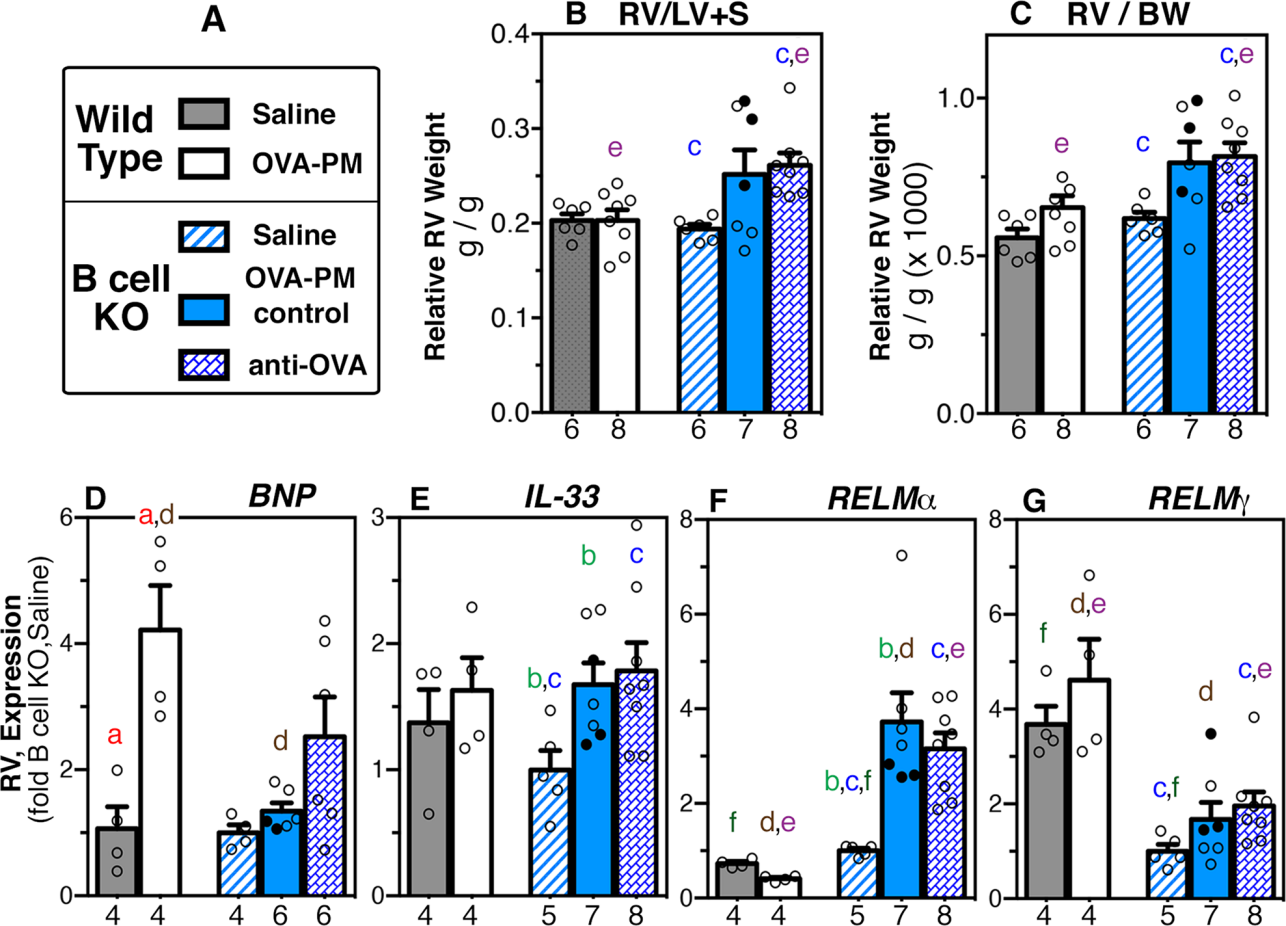
Right ventricular (RV) weight and RV-gene expression. Groups of wild type and B cell KO mice challenged with saline or OVA-PM were analyzed. Groups of OVA-PM challenged B cell KO mice were either controls [injected with control antibody (open circles) or given no injections (filled circles)] or injected with anti-OVA IgG1 antibody. **(A)** Legend. **(B-G)** Bar graphs show mean ± SEM and individual data for right ventricular weight calculated relative to **(B)** the weight of the left ventricle and septum (RV/LV+S), or **(C)** body weight (RV/BW); and gene expression in the right ventricle of [BNP **(D)**, IL-33 **(E)**, RELMα **(F)**, RELMγ **(G)**]. Gene expression in the lungs is shown as fold-increase over the means of the wild type saline group. Pairs of letters above the bars indicate the pairs of groups that showed significant differences (p<0.05) calculated with the Mann Whitney U test (unpaired, two-tailed). Numbers below the bars indicate the numbers of mice per group.

Right ventricular weight was measured as indicator of right ventricular hypertrophy [53] in the same animals studied previously (Fig 9A). As expected [16] wild type mice challenged with antigen and PM_2.5_ did not show an increase in right ventricular weight (Fig 9B and 9C). In contrast, antigen and PM_2.5_ challenged B cell KO mice that were injected with antigen-specific IgG1 had significantly increased right ventricular weights (Fig 9B and 9C). The significance levels (Mann-Whitney two-tailed test) for the comparison of the right ventricular weight relative to the weight of the left ventricle and septum for the group of OVA-PM_2.5_ B cell KO mice injected with antigen-specific IgG1 were p = 0.0007 relative to B cell KO saline, and p = 0019 relative to wild type, OVA-PM_2.5_.

To test the idea that exposure to antigen and PM_2.5_ induced distinct molecular programming in the right ventricle of B cell KO mice, expression of brain natriuretic peptide (BNP) as marker for right ventricular strain in humans [3] was examined together with IL-33, a mediator of communication between cardiac myocytes and fibroblasts [54], and RELMα and RELMγ. The latter cytokines are known for their regulatory effects in inflammation [55–58], proliferation of vascular cells [59, 60], and metabolism [61–63]. The expected [16] significant increase in BNP expression in the right ventricles of antigen and PM_2.5_ exposed wild type mice was not seen in B cell KO mice (Fig 9D). However, the right ventricular BNP levels in the group of antigen and PM_2.5_ exposed B cell KO mice injected with antigen specific IgG1 antibodies was not different the challenged wild type mice (Fig 9D). Relative to the saline exposed group, right ventricular expression of *IL-33* and *RELMα* was significantly increased in groups of antigen and PM_2.5_ exposed B cell KO mice, but not in wild type mice (Fig 9E and 9F). Only antigen and PM_2.5_ exposed B cell KO mice injected with antigen specific IgG1 showed a significant increase in right ventricular expression of *RELMγ* over the saline group (Fig 9G). B cell KO mice at baseline (saline group) differed from the saline-wild type group in the right ventricular expression of the RELM molecules (*RELMα* increased, *RELMγ* decreased, Fig 9F and 9G). Further, in the B cell KO mice the significant difference in RELM molecule expression was still apparent following exposure to antigen and PM_2.5_ (Fig 9F and 9G). The expression of *RELMβ*, the third member of the RELM molecule family in mice, was not detectible in the right ventricle.

## Discussion

Our study was aimed to understand the function of B cell responses and IgG1 antibody directed against exogenous antigen for the pulmonary hypertension phenotype. For that goal, antigen-primed animals were challenged intranasally with antigen together with PM_2.5_ to induce the pulmonary hypertension phenotype. Surprisingly, sensitization of B cell KO mice followed by challenge with antigen and PM2.5 did not elicit increased right ventricular systolic pressures. This response was fully restored in sensitized B cell KO mice that were reconstituted with antigen specific IgG1 during the intranasal challenge phase with antigen and PM_2.5_. Similar to our data showing exacerbating effects of IgG1, antigen-specific IgG1 administration has been reported to enhance antigen-induced airway hyperreactivity in antigen challenged mice [64]. The mechanism could be that IgG1 enhances cross-cellular antigen transport across airway epithelial cells via FcRn, thereby increasing the amount of antigen that is delivered to antigen presenting cells in the airways [65]. Further, antigen-specific IgG1 elicited by Th2 responses can have anaphylactic activity by binding to FcγRIII [66]. Both mechanisms could cause an exacerbated T cell and inflammatory response with increased cytokine production in the lung tissues of B cell KO mice injected with antigen specific IgG1. However, our examination of the immune response in B cell KO mice would argue against this mechanism of action of antigen-specific IgG1.

All markers of Th2-inflammation were intact in B cell KO mice challenged with antigen and PM_2.5_ independently of the presence of IgG1. MHCII expression by CD11c+ cells in the airways of naïve mice predicts the levels of IgG1 antibody production to a subsequent challenge with antigen [44]. Mouse RELMβ (that has no clear human homolog) induces inflammation, mucus cell hyperplasia, and peribronchial / perivascuar collagen deposition in mice [55]. IL-33 is an alarmin that induces Th2 responses, including, in a feed-forward loop, IL-13 [67], RELMα and RELMβ.

In contrast, B cell KO mice had defective IL-17A dependent inflammatory responses to challenge with antigen and PM_2.5_, although the numbers of Th17 cells in the lymph nodes and IL-17A and IL-17F expression in the lungs were at wild-type levels. This deficiency in the B cell KO mice was not corrected by reconstitution with antigen-specific IgG1. B cell KO mice have been studied previously for IL-17A induced inflammation in Pneumococcal infections. That study showed that B cells and antibody were dispensable for the protective IL-17A-directed and T cell regulated inflammatory responses [68]. The distinct inflammation inducing agents, proliferating bacteria [68] vs. soluble, non-replicating antigen and PM_2.5_ (this study) could explain why antigen-specific IgG1 is necessary and non-redundant for some but not all types of IL-17A-regulated inflammation. The significantly decreased inflammatory infiltrates in the lungs of all groups of B cell KO mice challenged with antigen and PM_2.5_ likely reflect the absence of B cells and the significantly decreased numbers of neutrophils in the lungs because of the decreased Th17-effector responses in these mice.

Our previous studies showed that the severe, irregular wall thickening with smooth muscle actin positive cells of small (<100μm) pulmonary arteries in response to exposure with antigen and PM_2.5_ was completely blocked by the neutralization of IL-13 [16]. The severe arterial thickening that developed in B cell KO mice exposed to antigen and PM_2.5_ was not different from the remodeling seen in wild type mice. The finding is in keeping with the intact, or even exacerbated IL-13-dependent inflammatory response in the B cell KO mice exposed to antigen and PM_2.5_. Likely downstream mediators of the severe pulmonary arterial thickening response in B cell KO mice include *MMP12* and the excessively expressed *RELMα* relative to wild type. MMP12 [69, 70], a metalloelastase, and other proteinases [71–73] are thought to be critical for the dissolution of the elastic lamina that allows for the migration and expansion of smooth muscle cells in the wall of the small pulmonary arteries. Mouse RELMα, the homolog of human RELMβ, is a mitogen for smooth muscle cells [59, 60] and can induce the pulmonary hypertension phenotype [58, 74]. RELMα also has potent anti-inflammatory effects [56, 57]. Future studies will need to be designed to test if the excessive expression of *RELMα* in the lungs could be one mechanism for the decreased IL-17A-dependent inflammatory markers in the lungs of antigen and PM_2.5_ exposed B cell KO mice.

The significantly increased right ventricular weight in the B cell KO mice injected with antigen-specific IgG1 during challenge with the antigen and PM_2.5_ mirrored the increase in right ventricular systolic pressure in the same animals. Future studies will need to examine the possibility that IgG1 specific to exogenous antigen together with PM_2.5_ has direct effects on pulmonary vascular endothelial cells as well as endocardial cells that result in increased vascular constriction. While the literature supporting detrimental effects of urban pollution on cardiovascular health including the right ventricle is growing [5, 11, 75, 76], the mechanism remains to be elucidated. Our current study advances this field by identifying non-redundant roles for B cells in directing the inflammatory response, and for antigen specific IgG1for the increase in RVSP and molecular changes in the right ventricle.

Our data surprisingly revealed a homeostatic role of B cells for the molecular responses in the right ventricle, particularly for RELM family members (RELMα, RELMγ). Compared to wild type, the B cell KO mice showed deregulated expression in the right ventricle of RELMα, RELMγ and IL-33. It is known that IL-33 can provide for homeostatic communication between cardiomyocytes and cardio-fibroblasts [54]. RELMα has been reported as a binding partner for Bruton’s tyrosine kinase [77] which is a critically important signaling molecule for B cells. However, it is not known if B cells produce or stimulate the production of RELMα or RELMγ. Furthermore, the roles of RELMα and RELMγ and their potential interactions with IL-33 and B cells for right ventricular function are currently not known and require further study.

Future studies aimed at understanding the signaling networks in endothelial, smooth muscle, fibroblasts, and cardiac cells are necessary to understand the mechanism by which the immune factors exert their effects. Receptors for IL-13 and IgG are present on these cell types. Signals via signal transducer and activator of transcription 6 (STAT6, IL-13-receptor initiated) and nuclear factor of activated T cells (NFAT, IgG–Fc-receptor initiated) can for example function synergistically for inflammatory cytokine production [78]. Several lines of evidence indicate that NFAT signaling can contribute to the behavioral changes in endothelial cells and smooth muscle cells that cause pulmonary arterial remodeling and constriction then resulting in pulmonary hypertension [17, 79, 80].

Because of the inherent limitations of animal experimentation, our study will require follow-up work to understand its relevance for the human condition. Agents with broad immune-suppressive action have been tried for the management of pulmonary hypertension with some success [81]. However, the studies were not directed against specific immune mediators such as antigen specific IgG1. Therefore, the effectiveness of interventions targeting specific mediators of the immune response matched to the individual pulmonary hypertension phenotype in humans remains to be determined.
